# Time dependence of ^137^Cs contamination in wild Japanese monkeys after the Fukushima Daiichi nuclear accident

**DOI:** 10.1007/s11356-022-23707-0

**Published:** 2022-10-29

**Authors:** Shin-ichi Hayama, Aki Tanaka, Setsuko Nakanishi, Fumiharu Konno, Yoshi Kawamoto, Kazuhiko Ochiai, Toshinori Omi

**Affiliations:** 1 Faculty of Veterinary Medicine, Nippon Veterinary and Life Science University, Musashino, Tokyo 180-8602 Japan; 2 Fukushima Mirai Agricultural Cooperative, Fukushima, 960-0185 Japan; 3 Tohoku Wildlife Management Center, Sendai, Miyagi 989-3212 Japan; 4 Primate Research Institute, Kyoto University, Inuyama, Aichi Japan

**Keywords:** ^137^Cesium, Fukushima Daiichi nuclear accident, Japanese monkeys, Muscle concentration, Transfer factor

## Abstract

Over the 10 years immediately after the Fukushima Daiichi nuclear accident, we measured the changes in the muscle ^137^Cs concentration (Bq/kg) of wild Japanese monkeys living in Fukushima City, which is located approximately 70 km from the Fukushima Daiichi Nuclear Power Plant. The muscle^137^Csconcentration, which was observed at a maximum of 13,500 Bq/kg immediately after the accident, had decreased to several hundred Bq/kg 10 years later. The muscle ^137^Cs concentration was significantly related to the soil contamination levels (10,000–30,000, 30,000–60,000, 60,000–100,000, and 100,000–300,000 Bq/m^2^), sex, age class (immature, mature), body weight (> 5000 g, 5000–10,000 g, < 10,000 g), and seasons (the cold period from December to April, the warm period from May to November).The value of muscle ^137^Cs concentration and the aggregated transfer factor (*T*_ag_: calculated by dividing muscle ^137^Cs concentration [Bq/kg] by soil ^137^Cs deposition density at the capture site [Bq/m^2^]) apparently decreased with time for several years. However, post hoc pairwise comparisons showed no difference from 2017 to 2020, and the accumulation of ^137^Cs in muscle may continue for some time.

**Supplementary Information:**

The online version contains supplementary material available at 10.1007/s11356-022-23707-0.

## Introduction

More than 10 years have passed since the Fukushima Daiichi nuclear accident occurred in March 11, 2011. We have studied the radioactive exposure and its effect on the health of Japanese monkeys (*Macaca fuscata*) inhabiting Fukushima City, which is located approximately 70 km from the Fukushima Daiichi Nuclear Power Plant (Hayama et al. 2013, 2017; Ochiai et al. 2014; Omi et al. 2020). These Japanese monkeys are the first wild primates in the world to be exposed to radiation as a result of the nuclear accident. In the previous study (Hayama et al. 2013), we compared the means of muscle radiocesium (^134^Cs + ^137^Cs) concentration in monkeys captured at different areas with soil contamination levels between April 2011 and June 2012. However, no studies, including those by our research group, have reported the long-term changes in radiocesium concentration in wild primates.

Game animals such as wild boars, black bears, and copper pheasants are used for food, and so muscle radiocesium concentration of these animals has been measured continuously in Fukushima Prefecture and by surrounding local governments. The measurement data have been used to analyze the concentration and the aggregated transfer factor of radiocesium in game animals (Tagami et al. 2016; Nemoto et al. 2018; Anderson et al. 2019; Saito et al. 2020). The aggregated transfer factor (*T*_ag_) is defined as the ratio of the mass radioactive material density (Bq/kg) in a specified object to the unit area radioactive material density (Bq/m^2^) (International Atomic Energy Agency: IAEA 2010) and was calculated using the following equation in these studies: *T*_ag_ (m^2^/kg) = ^137^Cs concentration (Bq/kg) in muscle of animal/^137^Cs soil deposition density at the capture site (Bq/m^2^).Although *T*_ag_ values in wildlife should be considered as means of carrying out screening calculations (IAEA 2010), it enables direct comparisons of *T*_ag_ values between species with respect to temporal and spatial factors by the varying deposition rates considered (Tagami et al. 2016). There have been many similar studies after nuclear tests and the Chernobyl nuclear accident in Europe using game animals, particularly deer and wild boars (Zibold et al. 2001; Ǻhman 2007; Strebl and Tataruch 2007; Semizhon et al. 2009; Gulakov 2014; Kapata et al. 2015). However, to our knowledge, there are no such studies on wild primates.

Our research group had reported the health effects of the Fukushima nuclear accident on Japanese monkeys, including a significant negative correlation between leukocyte depletion and muscle radiocesium concentration in immature individuals (Ochiai et al. 2014) as well as the delayed body weight growth rate and the small head size in fetuses conceived after the accident (Hayama et al. 2017). These health effects are thought to have a causal relationship with exposure dose, but these studies have not examined the relationship between individual exposure dose and health effects. Urushihara et al. (2018) and Endo et al. (2020) estimated the internal exposure dose of individual Japanese monkeys from the muscle radiocesium concentration of each individual. Semizhon et al. (2009) have concluded that the ^137^Cs levels found in wild boars have remained almost constant over the decade from 1998 to 2008 based on data from southern Germany. Gulakov (2014) also reported a similar behavior in accumulation of ^137^Cs in wild boars on contaminated areas around the Chernobyl exclusion zone. However, in Japanese monkeys, it is unclear how the concentration changes over time and what factors (e.g., season, sex, age, and body weight) affect the changes. Therefore, it is difficult to predict the future trends of the radiocesium concentration.

In order to evaluate the relationship between the exposure dose of radiation and health effects,it is inevitable to understand the time-dependent changes in muscle radiocesium concentration and affecting factors. In this study, we reported the measurement of^137^Cs concentration in the muscle tissue of Japanese monkeys in Fukushima City for the 10-year period from 2011 to 2020.We investigated the factors including age, sex, body weight, season, and the contamination level associated with^137^Cs concentration in the muscle tissue. We also evaluated *T*_ag_ for the same periods. Our goal was to investigate an effective indicator for Japanese monkeys that could predict future long-term exposure of the radiation.

## Methods

### Animals and ethics

The population of Japanese monkeys in Fukushima City has been systematically managed by Fukushima Prefecture to reduce damages to agricultural crops. Since 2008, our research group has been using the carcasses of captured individuals to study the population parameters, especially reproductive rates necessary for appropriate management of the Japanese monkey population (Hayama et al. 2011). Carcasses were provided by Fukushima City, with the permission of the governor of Fukushima Prefecture, in accordance with the Fukushima Japanese Monkey Management Plan (Fukushima Prefecture 2017), which was established under the Wildlife Protection and Hunting Management Law. The monkeys were captured using box traps and then euthanized with a gun by licensed hunters at the request of Fukushima City. The capture and euthanasia methods were in accordance with the guidelines of the management plan stated above and should not be an ethical concern. This euthanasia method was also in accordance with guidelines published by the Primate Research Institute, Kyoto University (2019). The Japanese monkeys inhabiting this area were not listed as an endangered species on the Japanese Red List, as revised by the Ministry of the Environment (2012).

In this study, carcasses of Japanese monkeys collected in Fukushima City between April 11, 2011 and December 25, 2020 were assessed. The dates and the locations of capture were recorded by the hunter who captured the animals. The carcasses were transported either frozen or refrigerated to Nippon Veterinary and Life Science University and promptly subjected to necropsy. The body weight of each monkey was measured in grams. During necropsy, the sex and the status of tooth eruption were checked, and 500–1000 g of muscle tissue was collected from the hind limbs for measuring^137^Cs concentration. The muscle tissue was stored frozen at – 30 °C until it was used to measure the radioactivity. The age of each animal was estimated from the status of tooth eruption, as described by Iwamoto et al. (1987), to divide the animals into the immature (0–4 years) and mature (≥ 5 years) age groups.

### Muscle^137^Cs concentration

The muscle samples in a half-thawed state were minced after removing connective tissue and fat as much as possible and then encapsulated in U8 containers (100 ml) or Marinelli containers (500 ml).

The radioactivity of ^137^Cs in the muscle samples was analyzed with a germanium semiconductor spectrometer (GC2020-7500SL-2002 CSL, Canberra, Meriden, CT) and a NaI (T1) scintillation detector (AT1320A, Atomtex, Minsk, Belarus). Data were corrected to a background radiation dose in the measurement environment on an as-needed basis. ^137^Cs concentration was determined by counting 661.6 keV gamma-ray emissions. The radioactivity of ^137^Cs was adjusted to the value on the day of capture based on its physical half-life (30.1 years). The limit of detection was set to about 5–10 Bq/kg. The muscle ^137^Cs concentration was calculated as the concentration of ^137^Cs per kilogram of fresh muscle.

We did not use a control in this paper because the radiocesium was below the detection limit from Aomori monkeys measured as a control in our previous study (Ochiai et al. 2014).

### Aggregated transfer factor (T_ag_)

*T*_ag_ (m^2^/kg) was calculated by dividing muscle ^137^Cs concentration (Bq/kg) by soil ^137^Cs deposition density at the capture site (Bq/m^2^). The soil ^137^Cs deposition density at the capture site (Bq/m^2^) for each monkey was estimated from the “Deposition of Radioactive Cesium of the Third Airborne Monitoring Survey (Decay correction: July 2, 2011)” by Japanese Ministry of Education, Culture, Sports, Science, and Technology (MEXT 2022; see the MEXT website in the references in this paper for details on how soil ^137^Cs deposition density was measured). The data consisted of radiocesium deposition densities at the median points of the quarter grid squares (approximately 250 × 250 m) defined by JIS X 0410.We used QGIS 2.16.1 to extrapolate the soil ^137^Cs deposition density at the capture site (referred to as “soil ^137^Cs deposition”in this paper). We calculated the soil ^137^Cs deposition value on the capture date for each monkey using the physical half-life and the time difference (days) between the capture date and the survey date (July 2, 2011).

The accuracy is higher in the method of calculating *T*_ag_ by actually measuring the concentration of radiocesium in the soil at each capture site than in the above method. However, this method is extremely costly, and it has been pointed out that neither method can be expected to have sufficient accuracy in wild animals with a wide home range (Anderson et al. 2019), so this method was not adopted in this study.

## Data analysis

**Fig. 1:**
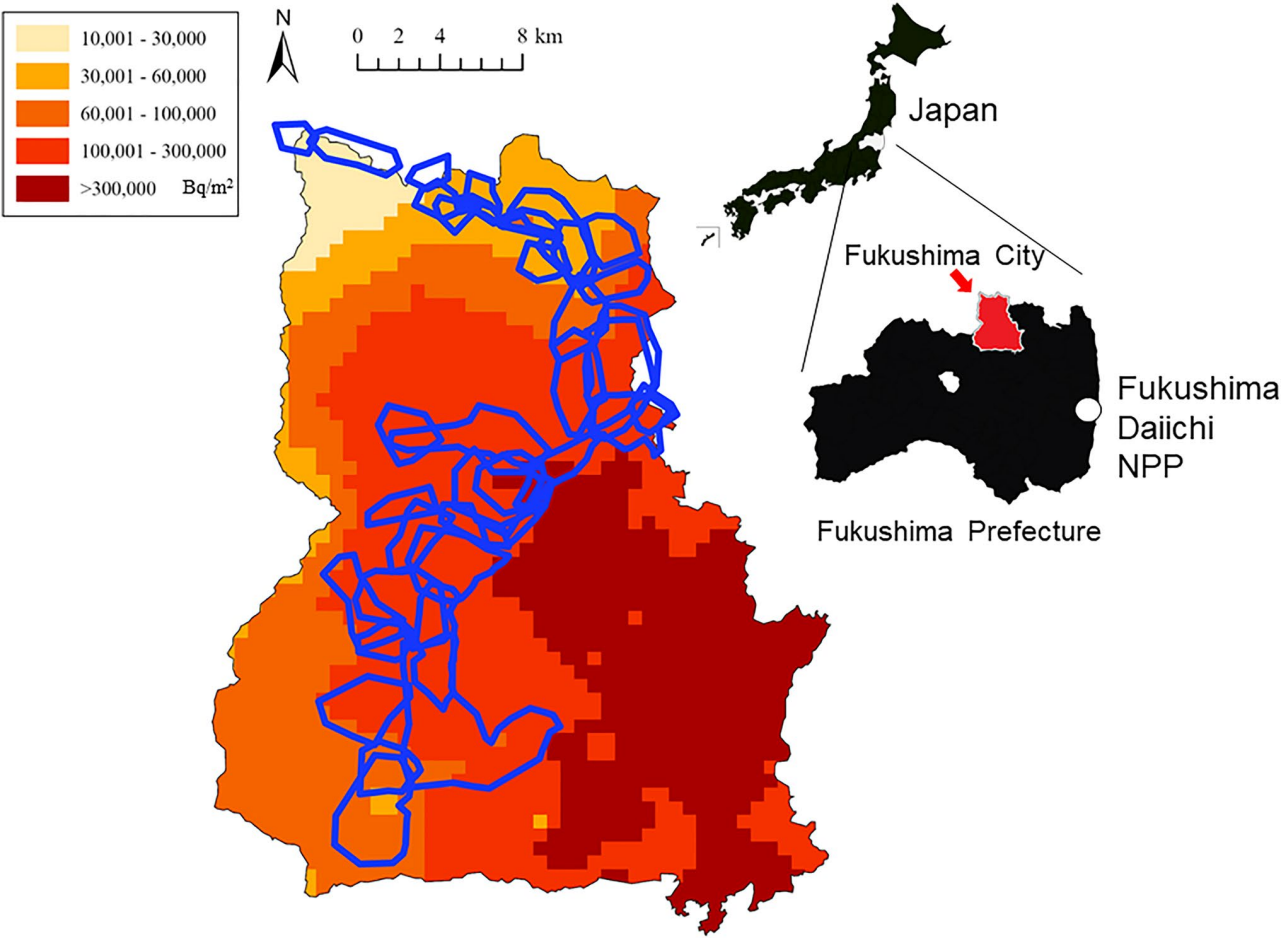
The soil contamination levels by radiocesium concentration (Bq/m^2^) and the distribution of monkey troops (irregular enclosed blue outlines) in Fukushima City. An upper right map showing the location of Fukushima City (red area), where the present investigation was conducted. This map was made according to the soil contamination map created by the Ministry of Education, Culture, Sports, Science, and Technology (converted to the values of July 2, 2011)

The Shapiro–Wilk test was performed to evaluate the normality of data for the continuous variables. Body weights by age (immature and mature) and sex (male and female) were reported as the median (range). Multiple non-parametric linear regression was performed to evaluate the association of muscle ^137^Cs concentration by year with age class (immature and mature), sex (male and female), season (the cold period, from December to April; and the warm period, from May to November, according to our previous paper; Hayama et al. 2013), body weight (> 5000 g, 5000–10,000 g, < 10,000 g), and the soil contamination level (10,000–30,000, 30,000–60,000, 60,000–100,000, and 100,000–300,000 Bq/m^2^)by radiocesium deposition density on July 2, 2011,depending on the capture site (Fig. 1), following the method in our previous paper (Hayama et al. 2013).

Univariate non-parametric linear regression was performed to evaluate the association of muscle ^137^Cs concentration and *T*_ag_ by year, and post hoc pairwise comparison was performed to investigate the difference in each year.

Stata statistical software: Release 16 (StataCorp, College Station, TX) was used in all analyses. For statistical estimation and inference, two-sided hypotheses and tests were used with a 5% significance level.

## Results and discussion

The study included 1459 Japanese monkeys, and the characteristics of the study population are described in Table 1.

**Table 1:** Characteristics of the study population of Japanese monkeys captured in Fukushima Prefecture in 2011–2020 (*n* = 1459)

Characteristics	Number of monkeys (%)
Year	
2011	86 (5.9)
2012	265 (18.2)
2013	141 (9.7)
2014	141 (9.7)
2015	114 (7.8)
2016	92 (6.3)
2017	122 (8.4)
2018	156 (10.7)
2019	209 (14.3)
2020	133 (9.1)
Body weight	
< 5000 g	568(38.9)
5000–10,000 g	498(34.1)
> 10,000 g	393(26.9)
Season	
Cold period	519 (35.6)
Warm period	940 (64.4)
Sex	
Female	711 (48.7)
Male	748 (51.3)
Age	
Immature	741 (50.8)
Mature	718 (49.2)
Soil contamination level	
1	172 (11.8)
2	259 (17.8)
3	276 (18.9)
4	752 (51.5)

The median body weight (range) was 3170 (640–8325) g for immature females, 9538 (1352–13,565) g for mature females, 3848 (504–8735) g for immature males, and 11,760 (1027–17,747) g for mature males.

### Muscle ^137^Cs concentration

**Fig. 2:**
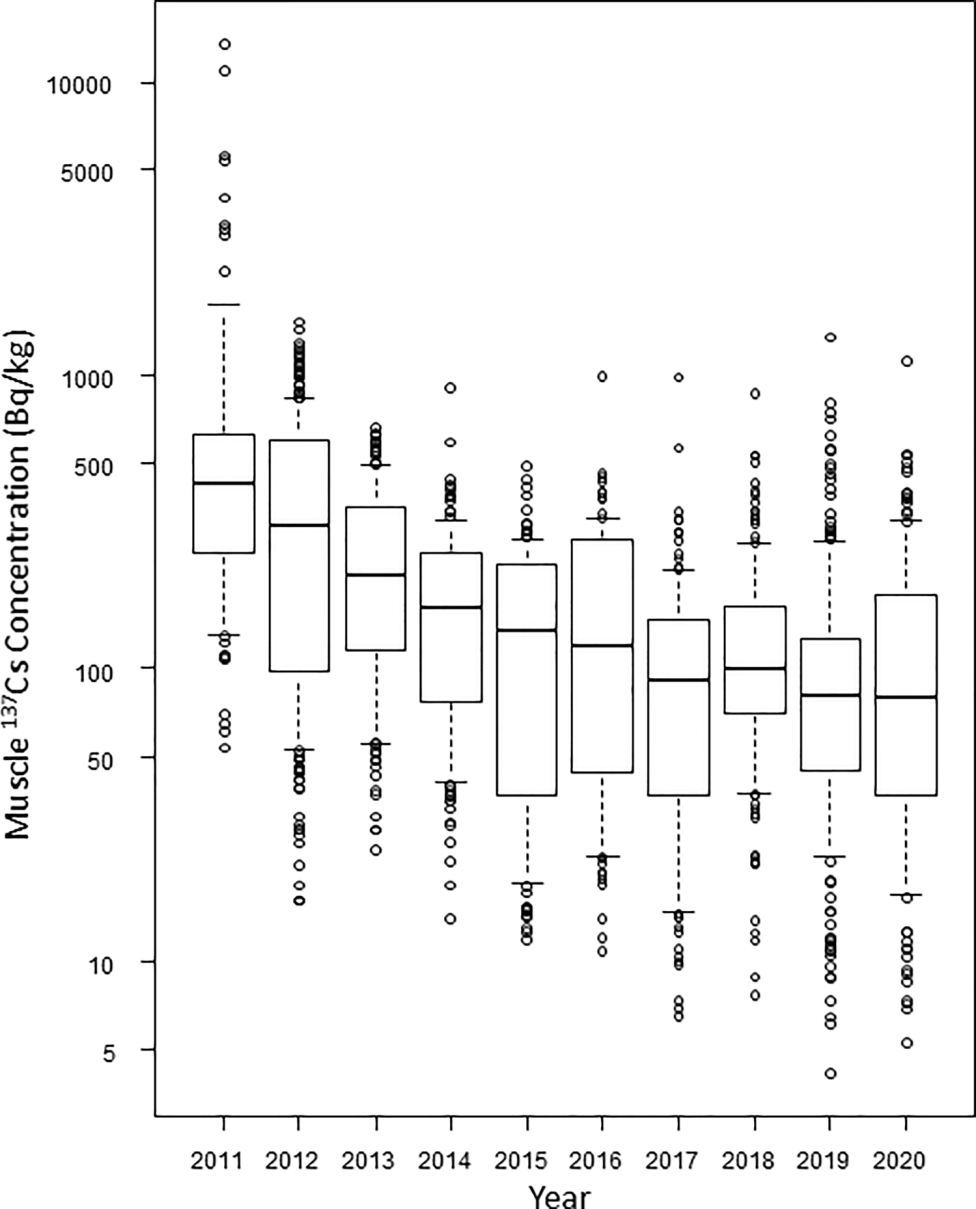
Muscle ^137^Cs concentration by year (2011–2020) in Japanese monkeys (*n* = 1459). The box plot shows 5th (lower whisker), 25th (bottom edge of the box), 75th (top edge of the box), and 95th (upper whisker) percentiles. The median concentrations are given as the line within the box and the open circles are outliers

This study was the first to observe long-term changes in radioactive cesium accumulation levels in wild primates, including fallout from nuclear tests. The muscle ^137^Cs concentrations apparently decreased with time for several years (Fig. 2) and were significantly reduced in subsequent years compared to 2011 (Table 2).However, post hoc pairwise comparisons showed no difference from 2017 to 2020, consecutively (Table 3). Even before 2017, there were no significant differences between 2014 and 2016, 2015 and 2016, 2018 - 2020, and 2016 and 2018, –2020 (Table 3). This may be due to the 95% confidence interval of the mean for 2016 being elevated above the before and after trend (Fig. 2), which may have caused some bias in the sample. In any case, the decreasing trend in muscle ^137^Cs concentrations may have decayed from around 2015 to 2016, and no significant decrease was observed after 2017.

**Table 2:** Univariate non-parametric linear regression for the muscle ^137^Cs concentration and year in Japanese monkeys captured in Fukushima Prefecture in 2011–2020 (*n* = 1459)

Year	Effect	95% CI			*P*-value
2011	Reference				
2012	− 567.41	− 997.59	to	− 137.24	0.01
2013	− 707.64	− 1136.93	to	− 278.35	0.001
2014	− 776.46	− 1205.40	to	− 347.52	< 0.0001
2015	− 814.05	− 1242.94	to	− 385.16	< 0.0001
2016	− 791.79	− 1221.36	to	− 362.23	< 0.0001
2017	− 843.11	− 1272.04	to	− 414.18	< 0.0001
2018	− 818.74	− 1247.57	to	− 389.92	< 0.0001
2019	− 830.43	− 1259.38	to	− 401.48	< 0.0001
2020	− 819.15	− 1248.35	to	− 389.96	< 0.0001

*CI*, confidence interval

**Table 3:** *P*-values of post hoc pairwise comparison for non-parametric regression of the muscle ^137^Cs concentration of Japanese monkeys captured in Fukushima Prefecture in 2011–2020 compared by year (*n* = 1459)

Year	2011	2012	2013	2014	2015	2016	2017	2018	2019
2012	**0.01**								
2013	** < 0.0001**	** < 0.0001**							
2014	** < 0.0001**	** < 0.0001**	** < 0.0001**						
2015	** < 0.0001**	** < 0.0001**	** < 0.0001**	**0.011**					
2016	** < 0.0001**	** < 0.0001**	** < 0.0001**	0.425	0.239				
2017	** < 0.0001**	** < 0.0001**	** < 0.0001**	** < 0.0001**	**0.048**	**0.007**			
2018	** < 0.0001**	** < 0.0001**	** < 0.0001**	**0.003**	0.736	0.146	0.087		
2019	** < 0.0001**	** < 0.0001**	** < 0.0001**	** < 0.0001**	0.27	**0.045**	0.403	0.416	
2020	** < 0.0001**	** < 0.0001**	** < 0.0001**	**0.012**	0.758	0.185	0.156	0.98	0.507

*P*-values for combinations with significant differences are indicated in bold

Season, sex, age, body weight, and the soil contamination level were significant factors associated with muscle ^137^Cs concentration in monkeys (Table 4, Figs. S1–S4). Our results indicated that the soil contamination level at the time of the accident in 2011 continued to affect the muscle ^137^Cs concentrations of monkeys thereafter until 2020.　In our previous paper (Hayama et al. 2013), we observed the transition of muscle radiocesium concentration in Japanese monkeys during the 15 months after the Fukushima accident and found that it increased in the cold period (December to April) than the warm period (May to November).In the present 10-year observation, an increase in muscle ^137^Cs concentration was also observed in the cold period (Table 4).Cases of seasonal increases in radiocesium concentrations in muscle in large mammals have been reported. For example, radiocesium concentrations in muscle were elevated during winter in wild boars and bears in Fukushima (Nemoto et al. 2018) and reindeer in Norway (Eikelmann et al. 1990), and during summer in wild boars in Germany (Hohmann and Huckschlag 2005), respectively. These elevations might be related to the feeding habits of wildlife in each region.

**Table 4:** Multiple non-parametric linear regression for the muscle ^137^Cs concentration and associated factors, including body weight, age, sex, season, and soil contamination levels in Japanese monkeys captured in Fukushima Prefecture in 2011–2020 (*n* = 1459)

Associated factors	Effect	95% CI	*P*-value
Body weight			
> 5000 g	Reference		
5000–10,000 g	78.07	23.06–133.08	0.005
< 10,000 g	111.53	28.50–194.56	0.008
Age			
Immature	Reference		
Mature	199.4	42.4–158.5	0.001
Sex			
Female	Reference		
Male	71.7	13.1–130.3	0.017
Season			
Warm period	Reference		
Cold period	73.1	23.7–122.6	0.004
Soil contamination level			
1	Reference		
2	73.3	23.3–123.4	0.003
3	128	104–152.0	< 0.0001
4	267.9	214.9–320.9	< 0.0001

*CI*, confidence interval

Japanese monkeys in snowy areas feed mainly on winter buds and the cambium layer of tree bark during winter (Watanuki and Nakayama 1993; Watanuki et al. 1994; Tsuji et al. 2006). It has been shown that radiocesium in the soil is taken up by trees and accumulates in their winter buds and the cambium layer at a relatively high concentration (Yoshida et al. 2011). Japanese monkeys feed on mushrooms and bamboo shoots in addition to bark and winter buds during the cold period (Koganezawa 1997; Ishii 2002), and cesium accumulated in relatively high concentrations in these foods (Komatsu et al. 2019; Higaki et al. 2012; Sasaki et al. 2015). Therefore, it was expected that the upward trend during the cold period would continue. Nemoto et al. (2018) investigated the seasonal variation of muscle ^137^Cs concentration in Asian black bears (*Ursus thibetanus*) and wild boars (*Sus scrofa*) in Fukushima Prefecture and suggested that the most important factors for evaluating a target species were feeding habits, habitat use, and seasonal changes in physiology.

The muscle ^137^Csconcentration was related to body weight, sex, and age class (Table 4).The body weight of Japanese monkeys used in this study ranged from 505 to 17,747 g, with a more than 30-fold difference between the maximum and minimum. The body weight was considered to be dependent on sex and age; therefore, it was unclear which factor most significantly affected the muscle ^137^Cs concentration in this study. There are no studies that have found a relationship between muscle radiocesium concentration and body weight over time in wild mammals. The differences in body weight may affect the balance between the intake and excretion of radiocesium. Higley et al. (2003) argued that the concentration of radioactive material accumulated in the animal body could be predicted by the metabolic rate and relative growth in animals, and that the biological half-life of an individual was related to body weight. In contrast, Beresford et al. (2004) concluded that further research was needed since there were several studies showing that the biological half-life of radiocesium was independent of body weight. The present study could not clarify the cause of the association of muscle ^137^Cs concentration on body weight.

### Aggregated transfer factor

**Fig. 3:**
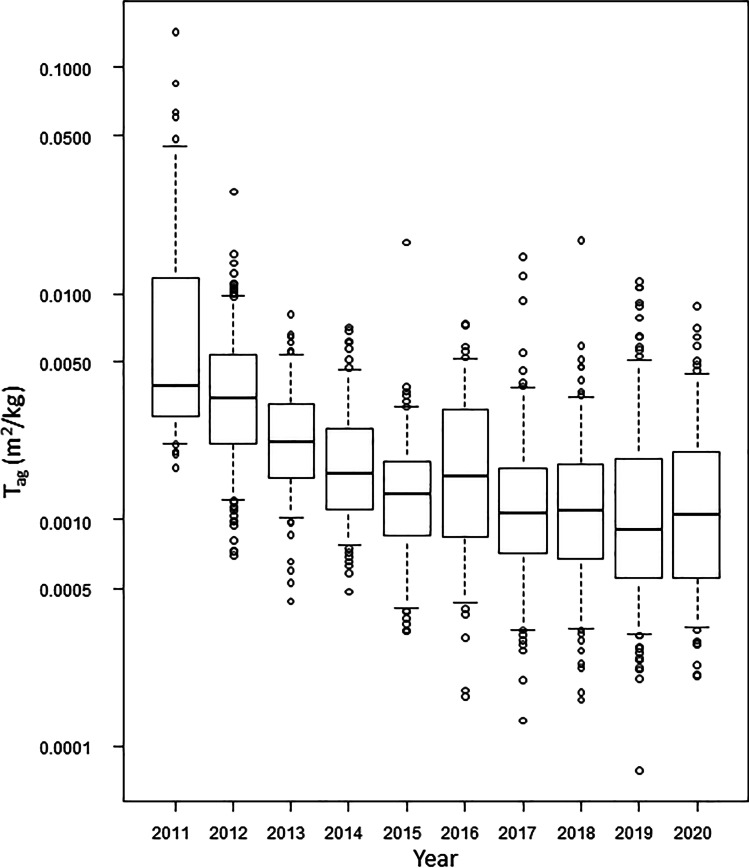
^137^Cs aggregated transfer factor [*T*_ag_ (m^2^/kg)] by year (2011–2020) in Japanese monkeys (*n* = 1459). The box plot shows 5th (lower whisker), 25th (bottom edge of the box), 75th (top edge of the box), and 95th (upper whisker) percentiles. The median *T*_ag_ is given as the line within the box, and the open circles are outliers

The aggregated transfer factor (*T*_ag_; m^2^/kg), which is the ratio of the soil ^137^Cs deposition to the muscle ^137^Cs concentration, was calculated, and the *T*_ag_ values apparently decreased with time for several years (Fig. 3) and were significantly reduced in subsequent years compared to 2011 (Table 5). However, post hoc pairwise comparisons showed no difference from 2017 to 2020, consecutively (Table 6). As mentioned above, the *T*_ag_ values showed a similar trend, as some years prior to 2017 did not show significant differences in the means of muscle ^137^Cs concentrations (*p*-values for years with significant differences are indicated in bold).

**Table 5:** Univariate non-parametric linear regression for ^137^Cs aggregated transfer factor [*T*_ag_ (m^2^/kg)] and year in Japanese monkeys captured in Fukushima Prefecture in 2011–2020 (*n* = 1459)

Year	Effect	95% CI			*P*-value
2011	Reference				
2012	− 0.00675	− 0.01103	to	− 0.00248	0.002
2013	− 0.00841	− 0.01268	to	− 0.00415	< 0.0001
2014	− 0.00891	− 0.01318	to	− 0.00465	< 0.0001
2015	− 0.00937	− 0.01364	to	− 0.00510	< 0.0001
2016	− 0.00888	− 0.01315	to	− 0.00461	< 0.0001
2017	− 0.00940	− 0.01368	to	− 0.00513	< 0.0001
2018	− 0.00945	− 0.01372	to	− 0.00518	< 0.0001
2019	− 0.00938	− 0.01365	to	− 0.00511	< 0.0001
2020	− 0.00934	− 0.01360	to	− 0.00507	< 0.0001

*CI*, confidence interval

**Table 6:** *P*-values for post hoc pairwise comparison for non-parametric regression of ^137^Cs aggregated transfer factor [*T*_ag_ (m^2^/kg)] of Japanese monkeys captured in Fukushima Prefecture in 2011–2020 compared by year (*n* = 1459)

Year	2011	2012	2013	2014	2015	2016	2017	2018	2019
2012	**0.002**								
2013	** < 0.0001**	** < 0.0001**							
2014	** < 0.0001**	** < 0.0001**	**0.001**						
2015	** < 0.0001**	** < 0.0001**	** < 0.0001**	**0.015**					
2016	** < 0.0001**	** < 0.0001**	**0.02**	0.869	**0.03**				
2017	** < 0.0001**	** < 0.0001**	** < 0.0001**	**0.017**	0.902	**0.03**			
2018	** < 0.0001**	** < 0.0001**	** < 0.0001**	**0.002**	0.7	**0.007**	0.821		
2019	** < 0.0001**	** < 0.0001**	** < 0.0001**	**0.005**	0.965	**0.016**	0.926	0.704	
2020	** < 0.0001**	** < 0.0001**	** < 0.0001**	**0.011**	0.852	**0.029**	0.759	0.528	0.798

*P*-values for combinations with significant differences are indicated in bold

*T*_ag_ for wild primates had not been reported previously, and this was the first to report for the long-term observation. Similar trends of time dependence in muscle ^137^Cs concentration and *T*_ag_ had been reported for roe deer and wild boars in Chernobyl and for wild boars and deer in Fukushima (Tagami et al. 2016; Strebl and Tataruch 2007; Gulakov 2014; Steinhauser and Saey 2016). Steinhauser and Saey (2016) discussed two possible reasons why there was no reduction in muscle ^137^Cs concentration in wild boars. Firstly, soil was not the only reservoir because there were intermediary organisms such as lichen or fungi that may act as soil-independent sources (secondary reservoirs) for the boars. Secondly, a partial transition of granular (i.e., insoluble) radiocesium to ionic water-soluble radiocesium might increase the bioavailability to fungi and fodder organisms of the boars and, hence, counterbalance the decline in absolute numbers of radiocesium atoms in the reservoir. As mentioned above, the monkeys in Fukushima also eat organisms with high radiocesium concentrations, so we considered that the same reasons given by Steinhauser and Saey (2016) could apply for the monkeys in Fukushima.

The maximum geometric mean value (0.0056 m^2^/kg in 2011) of *T*_ag_ by year in our study was approximately one-tenth that of animals in Chernobyl (Strebl and Tataruch 2007; Semizhon et al. 2009; Kiefer et al. 1996) and similar to that of animals in Fukushima (Tagami et al. 2016; Nemoto et al. 2018) for large mammals such as wild boars and deer (Table 7). Konoplev et al. (2016) suggested that the reason for the decline of radiocesium was due to different soil types in the Fukushima area compared to Chernobyl. Onda et al. (2020)reported that anthropogenic activities, high run-off, and steep topography led to a rapid decline in the activity concentration of ^137^Cs in soils and rivers, especially in the first year after the accident, and the decline in exposed radioactivity was notably faster than that seen after the Chernobyl, likely related to differences in geography and climate and the intensive remediation activities in Fukushima. In fact, of the radiocesium that fell to the ground in Fukushima in 2011, 70% was deposited on tree trunks in cedar forests and 77% in the deciduous layer in deciduous broadleaf forests, while in 2018, 94% in cedar forests and 73% in deciduous forests were deposited in soil (Onda et al. 2020). Values of *T*_ag_ presented in Table 7 estimated soil deposition of ^137^Cs from physical decay, so it was expected that the amount of ^137^Cs actually available to herbivores in Fukushima would be much smaller than that. Such a background may make the value of *T*_ag_ in wild animals in Fukushima relatively smaller than that in Chernobyl.

**Table 7:** Geometric means of ^137^Cs aggregated transfer factor [*T*_ag_ (m^2^/kg)] for wild mammals after fallout in the Fukushima and Chernobyl nuclear accidents

Accident	Species	Years	Investigation site	*T*_ag_ (m^2^/kg)	Source
Chernobyl	Roe deer (*Capreolus capreolus*)	1986–2003	Weinsberger Forest, Austria	0.046–0.008	Strebl and Tataruch (2007)
		1986–2000	Kobernausser Forest, Austria	0.094–0.016	
		1987–1991	Southern Germany	0.25–0.0002	Kiefer et al. (1996)
	Red deer (*Cervus elephus*)	1988–1989	Kobernausser Forest, Austria	0.028–0.007	Strebl and Tataruch (2007)
	Wild boar (*Sus scrofa*)	1987–2003	Weinsberger Forest, Austria	0.046–0.004	
		1988–2000	Kobernausser Forest, Austria	0.159–0.031	
		2003–2004	Ravensburg, Southern Germany	0.062–0.008	Semizhon et al. (2009)
Fukushima	Asian black bear (*Ursus thibetanus*)	2011–2015	Fukushima, Miyagi, Ibaraki, Tochigi, and Gunma, Japan	0.0052–0.0028	Tagami et al. (2016)
		2011–2016	Fukushima, Japan	0.0022 (all years)	Nemoto et al. (2018)
	Wild boar (*Sus scrofa*)	2011–2015	Fukushima, Miyagi, Ibaraki, Tochigi, and Gunma, Japan	0.0068–0.0026	Tagami et al. (2016)
		2011–2016	Fukushima, Japan	0.0032 (all years)	Nemoto et al. (2018)
	Sika deer (*Cervus nippon*)	2011–2015	Fukushima, Miyagi, Ibaraki, Tochigi, Gunma, Japan	0.0072–0.0051	Tagami et al. (2016)
	Japanese monkey (*Macaca fuscata*)	2011–2021	Fukushima City, Japan	0.0056–0.0011	Present study

## Conclusion

Our results indicated that the amount of ^137^Cs accumulated in muscle and the transfer factor apparently decreased with time for several years. The results showed that the amount of ^137^Cs accumulated in muscle and the transfer coefficient clearly decreased over time in the years following the accident; however, there was no significant difference between years after 2017. It is well known that temporal changes of radiocesium (excluding physical decay) almost “level off,” especially in semi-natural and natural ecosystems (Smith et al. 1999, 2000). Presumably, radiocesium could continue to accumulate in Japanese monkeys for time being, and there was concern that the effects of low-dose exposure might persist.

However, the results of this study indicate that radio cesium may continue to accumulate in Japanese monkeys for some time to come, which raised concern that the effects of low-dose exposure of radiation persist. Internal exposure could be estimated from the amount of ^137^Cs accumulated in the body, and the relationship between the exposure dose and its health effects should be reexamined in the future study. It is important to keep on monitoring the changes in the amount of ^137^Cs accumulated in Japanese monkeys over time and investigate the factors that might affect the internal exposure.

## Supplementary Information

Below is the link to the electronic supplementary material.

Supplementary file1 (DOCX 465 KB)

## Data Availability

The data supporting the findings of this study are available from the corresponding author, Shin-ichi Hayama, upon request.
