# Supplementary material for: Time dependence of 137Cs contamination in wild Japanese monkeys after the Fukushima Daiichi nuclear accident
Source: Environ Sci Pollut Res Int. 2022 Oct 29;29(58):88359–68. doi: 10.1007/s11356-022-23707-0 (PMC9672003; doi:10.1007/s11356-022-23707-0)
Supplement: Supplementary file 1 — Supplementary file1 (DOCX 465 KB) [file 11356_2022_23707_MOESM1_ESM.docx]

*Supplementary Material*

Time dependence of ^137^Cs contamination in wild Japanese monkeys after the Fukushima Daiichi nuclear accident

Shin-ichi Hayama*, Aki Tanaka*, Setsuko Nakanishi*, Fumiharu Konno^§^, Yoshi Kawamoto*, Kazuhiko Ochiai*, Toshinori Omi*.

*: *Faculty of Veterinary Medicine, Nippon Veterinary and Life Science University, Musashino, Tokyo, Japan;*

§: *Tohoku Wildlife Management Center, Sendai, Miyagi, Japan*

Email: [*hayama@nvlu.ac.jp*](mailto:hayama@nvlu.ac.jp)

**Contents**

**Fig S1** Association between ^137^Cs concentration in muscle and body weight (L:>5,000g, M: 5,000-10,000g, H: <10,000g).

**Fig S2** Association between ^137^Cs concentration in muscle and sex (Female and Male) /age class (Immature and Mature).

**Fig S3** Association between ^137^Cs concentration in muscle and season (Cold period from December to April and Warm period from May to November).

**Fig S4** Association between ^137^Cs concentration in muscle and soil contamination level (10,000–30,000, 30,000–60,000, 60,000–100,000, and 100,000–300,000 Bq/m^2^)

**
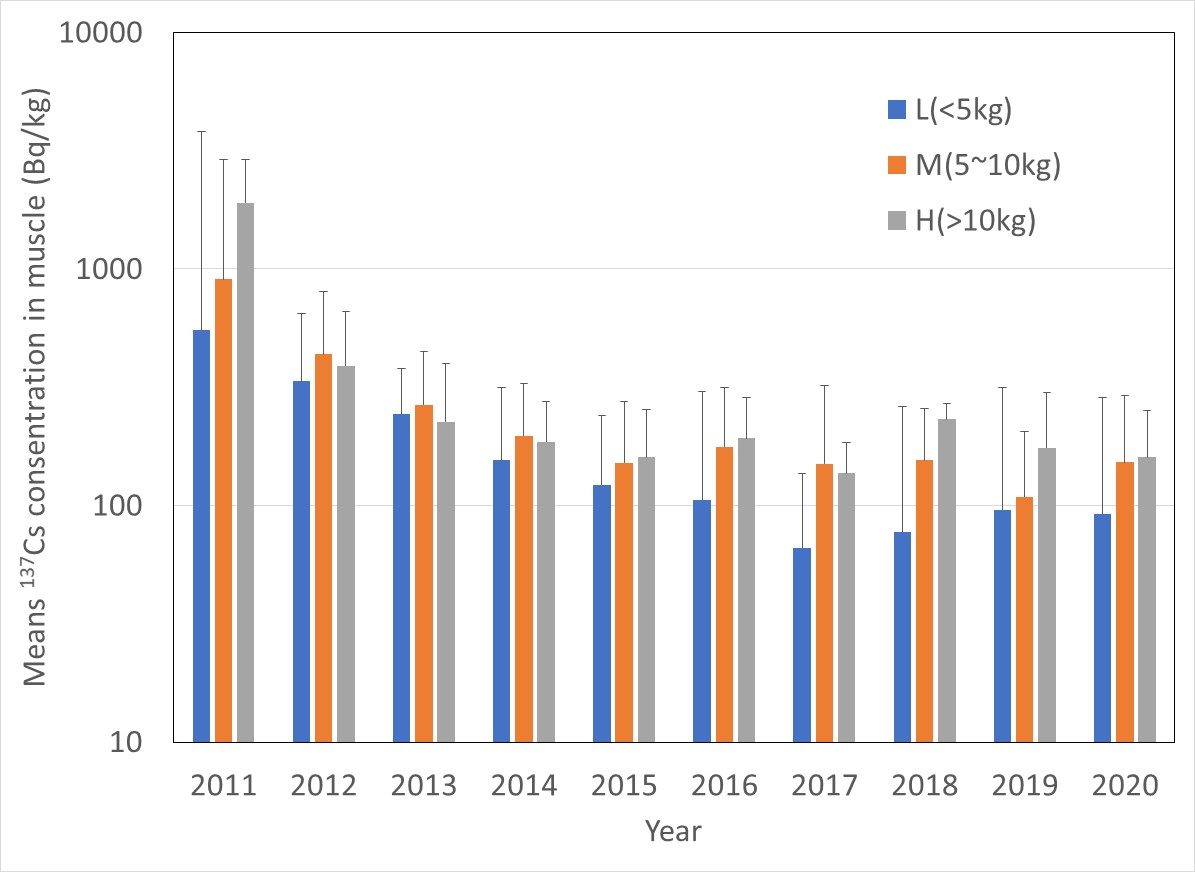
**

**Fig S1** Association between ^137^Cs concentration in muscle and body weight (L:>5,000g, M: 5,000-10,000g, H: <10,000g).

Error bar exhibit standard deviation.

**
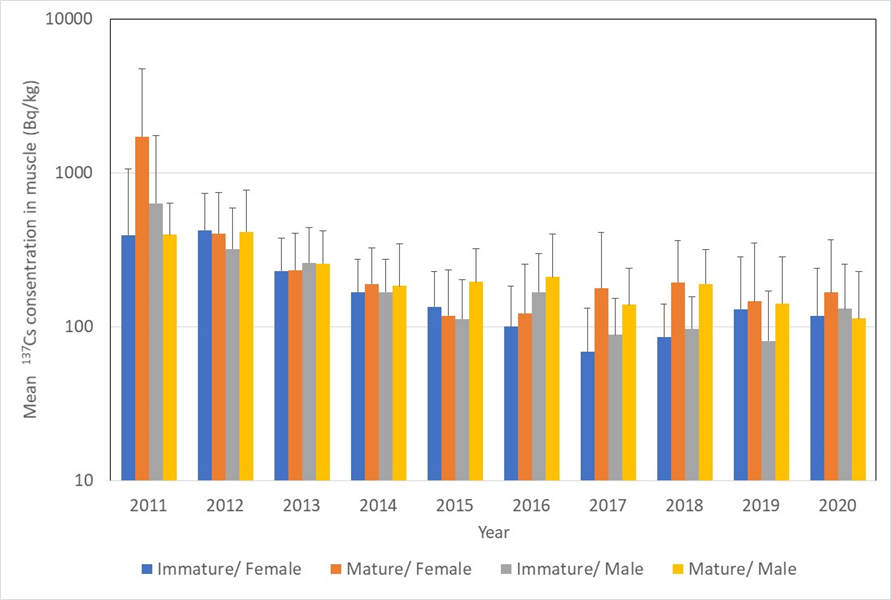
**

**Fig S2** Association between ^137^Cs concentration in muscle and sex (Female and Male) /age class (Immature and Mature).

Error bar exhibit standard deviation.

**
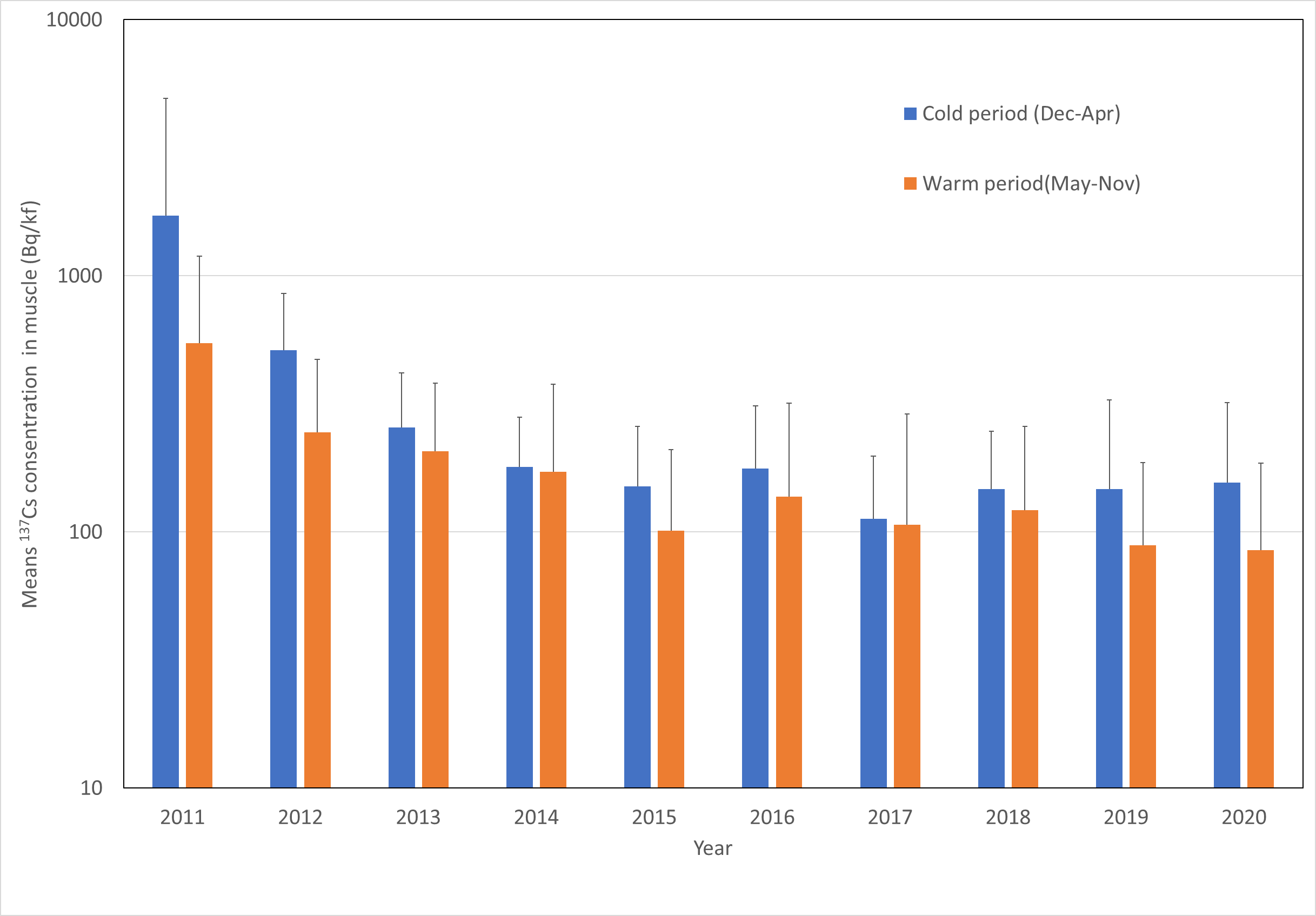
**

**Fig S3** Association between ^137^Cs concentration in muscle and season (Cold period from December to April and Warm period from May to November).

Error bar exhibit standard deviation.

**
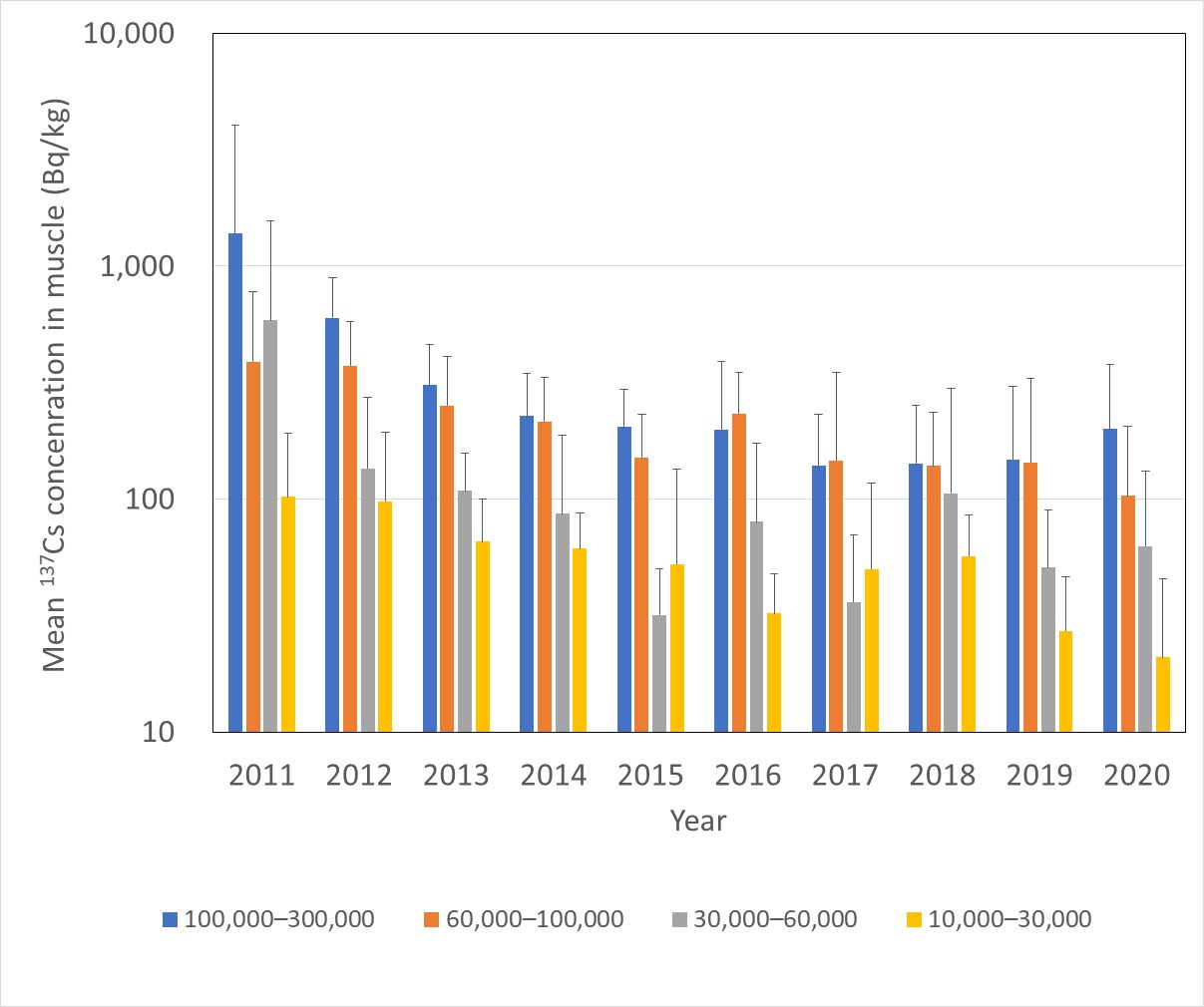
**

Soil contamination levels at capture sites (Bq/m^2^)

**Fig S4** Association between ^137^Cs concentration in muscle and soil contamination level (10,000–30,000, 30,000–60,000, 60,000–100,000, and 100,000–300,000 Bq/m^2^)

Error bar exhibit standard deviation.
